# The effect of guest cations on proton conduction of LTA zeolite†

**DOI:** 10.1039/d0ra09917a

**Published:** 2021-01-29

**Authors:** Huaizhong Shi, Jiani Zhang, Jiyang Li

**Affiliations:** State Key Laboratory of Inorganic Synthesis & Preparative Chemistry, College of Chemistry, Jilin University Changchun 130012 P. R. China lijiyang@jlu.edu.cn

## Abstract

Zeolites as important crystalline microporous materials have been widely used as catalysts, sorbents and ion-exchangers. In such materials, the guest ions in the pores can not only balance the charge of the framework but also make a difference to the pore environment and the resulting performance. In this work, we focus on the proton conduction properties of zeolites, and have comprehensively studied for the first time the effect of guest ions on proton conduction. To this end, aluminosilicate NaA zeolite (LTA) and ion-exchanged NaA zeolites by different guest ions (*i.e.* Li^+^, K^+^, Mg^2+^, Ca^2+^ and Sr^2+^) have been successfully synthesized. The study indicates that the guest ions could affect the proton conduction properties through the synergistic effect between the pore features (*e.g.* pore size, pore polarity, *etc.*) and guest ions, the ionic concentrations, and the interference between different ions. Among various guest ions, the existence of Na^+^ ions can greatly promote the proton conduction properties. The proton conductivity of NaA can reach 1.98 × 10^−3^ S cm^−1^ (100% RH) at room temperature and 9.12 × 10^−3^ S cm^−1^ (80 °C) under the condition of 100% RH. In addition, guest monovalent ions (Li^+^, Na^+^ and K^+^) exhibit better proton conductivity than divalent ions (Mg^2+^, Ca^2+^ and Sr^2+^). The distinct effect of these guest ions enables zeolites with tunable proton conductivity, which will provide more opportunities to design zeolitic proton conducting materials.

## Abstract 2

The as-synthesized NaA zeolite with the proton conductivity of 9.12 × 10^−3^ S cm^−1^ (80 °C, 100% RH) can show unique cations effects on proton conduction property with different guest cations substituted (*e.g.* Li^+^, K^+^, Mg^2+^, Ca^2+^ and Sr^2+^).

## Graphical abstract

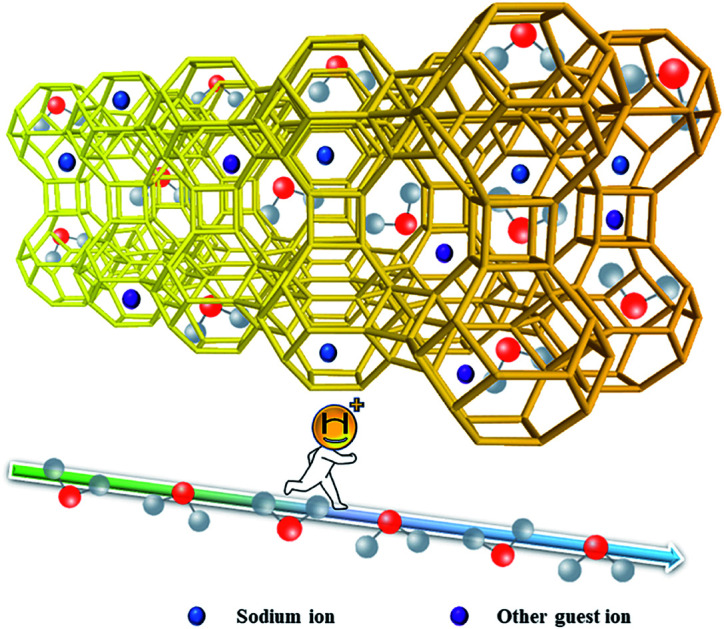

## Introduction

Facing increasing energy and environmental issues, it is highly desired to explore new energy materials to meet the demands of production and lives.^1–5^ In recent years, the development of proton exchange membrane fuel cells (PEMFCs) has aroused great interest. In such fuel cells, proton conductivity is a key parameter for the performance of solid electrolytes.^6–12^ Recently, metal–organic frameworks (MOFs) as emerging proton conducting materials have caught people's attention.^13–17^ A series of MOFs with excellent proton conduction properties have been reported, such as the MIL-53, MIL-101, MOF-74, HKUST-1, ZIF-8, UIO-66 and BUT-8(Cr) families.^18–26^ Generally, water plays a vital role in the proton conduction of the MOFs. In the structures of MOFs, water molecules can make a difference to strengthen connections or interactions to form hydrogen bonding networks to realize proton transport channels.^15,27^

Zeolites are a class of inorganic microporous materials with ordered framework and channel structure, which have extensive applications in the fields of adsorption, separation, catalysis.^28–32^ As with MOFs, zeolites have good water adsorption capacity, thus they may be potential proton conduction materials. However, the study of zeolites in proton conduction is limited. Up to now, there are only a few reports on proton conduction property of zeolite materials (*e.g.* LTA, FAU, ZSM-5, Beta), and their proton conductivities usually reach the order of magnitude 10^−3^ to 10^−4^ S cm^−1^.^33–38^ Moreover, some of these reported zeolites have been applied in PEMFCs.^39,40^

Previous studies indicate that different cations (*e.g.* Na^+^, K^+^) and anions (Cl^−^, Br^−^) have distinct effects on the water dynamics, which can strengthen or weaken non-bonding interactions of water.^41–44^ Meanwhile, it suggests that the guest ions may have great influence on the performance of water-mediated proton conducting materials. For example, Dincă group reported a Cu-azolate MOF, which could uptake stoichiometric loading of metal halides (*e.g.* Li^+^, Mg^2+^ and Al^3+^) to modulate the ion-pairing strength, giving variable ionic conductivity and activation energy with the function of anion identity.^45^ Moreover, we have synthesized two zeolite-like aluminophosphates in the presence of alkali or alkaline earth metal cations (*i.e.* Mg^2+^, Na^+^), in which the co-existence of water molecules and guest metal cations in the pores can effectively enhance the proton conductivity of materials.^46,47^ However, the systematic study of the effect of various guest ions on the proton conduction of nanoporous materials is rare.

Zeolites, especially aluminosilicate zeolites, have ion-exchanged property. In this work, aluminosilicate NaA (LTA zeotype) zeolite is selected as a target to explore the effect of various guest cations on its proton conduction property. NaA zeolite with Si/Al ratio of 1, and several ion-exchanged zeolites including LiA, KA, MgA, CaA and SrA zeolites have been successfully synthesized. Meanwhile, the commercial LTA zeolites of 3A, 4A and 5A are utilized as a comparison. These zeolites have been fully characterized, and their proton conduction properties have been studied at varied temperature and humidity. The results demonstrate that the different guest ions could modulate the proton conductivity of zeolites. Such influence of guest ions based on the synergistic effect between the pore features (*e.g.* pore size, pore polarity, *etc.*) and guest ions, the ion concentration and the interference between different ions have been further elucidated.

## Experimental

### Reagents and apparatus

All the chemical reagents were purchased from commercial channels without further purification. And the commercial zeolites of 3A, 4A, 5A were brought from Tianjin Fuchen Chemical Reagents Factory.

### Synthesis of NaA zeolite

NaA zeolite was synthesized in Na_2_O–Al_2_O_3_–SiO_2_–H_2_O hydrothermal system. Typically, 0.09 g NaOH, 1.03 g NaAlO_2_ and 2.60 g Na_2_SiO_3_·9H_2_O were dissolved in 10 mL distilled water under the vigorous stirring for an hour. And then the obtained white emulsion was placed into 25 mL Teflon-lined stainless autoclave and crystalized at 100 °C oven for 4 h. After finishing crystallization, cooling the reaction product to room temperature. After then, washing the reaction product with distilled water under centrifugation until the eluate pH to 7–8 and then drying it at 90 °C oven overnight.

### Synthesis of ion-exchanged zeolites

Ion-exchanged zeolites were synthesized by soaking as-synthesized NaA zeolite into five different salt solutions of 1 mol L^−1^ LiNO_3_, 1 mol L^−1^ KNO_3_, 0.5 mol L^−1^ Mg(NO_3_)_2_, 0.5 mol L^−1^ Ca(NO_3_)_2_, and 0.5 mol L^−1^ Sr(NO_3_)_2_ for two hours under 70 °C water-bath heating and stirring conditions, twice. The obtained products were washed by distilled water five times under centrifugal conditions and dried at 90 °C oven overnight, and the final samples named LiA, KA, MgA, CaA, SrA, respectively. For comparison, KA-1 and CaA-1 with lower ion-exchange degree were synthesized by soaking the as-synthesized NaA into 1 mol L^−1^ KNO_3_ and 0.5 mol L^−1^ Ca(NO_3_)_2_ for 90 minutes and then washed with distilled water five times under centrifugal conditions and dried at 90 °C oven overnight.

### Characterizations

The phase purity of zeolites was determined by powder X-ray diffraction (PXRD) D/Max-2550. Morphology analysis was performed by scanning electron microscope (SEM) JSM-7800F. Water content of as-synthesized zeolites was estimated by thermogravimetry (TG) TGA Q500, and water vapor adsorption isotherms were performed by chemical adsorption instrument autochem II 2920. The elemental analysis was performed on Inductive Coupled Plasma Emission Spectrometer (ICP) iCAP 7600 ICP-OES. The samples were pressed into a small wafer with 1 cm in diameter under 12 MPa pressure for ten minutes. The proton conductivity was measured by using a double probe method on Solartron 1260 + 1287 and the frequency ranged from 1 Hz to 10^6^ Hz. The proton conductivity is calculated by the formula 
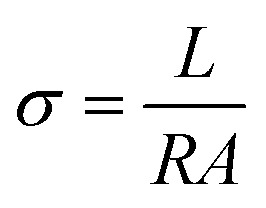
 where *σ* is proton conductivity (S cm^−1^), *L* is the thickness (cm) of the wafer sample, *R* is the resistance of the wafer sample (Ω), and *A* is the area of the circle (cm^2^). 
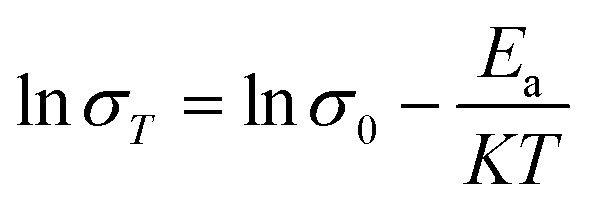
 where *σ* is proton conductivity (S cm^−1^), *K* is the Boltzmann constant (*K* = 8.6 × 10^−5^ eV K^−1^) and *T* is the temperature (K).

## Results and discussion

### Structure and characterization of NaA zeolite

**Fig. 1:**
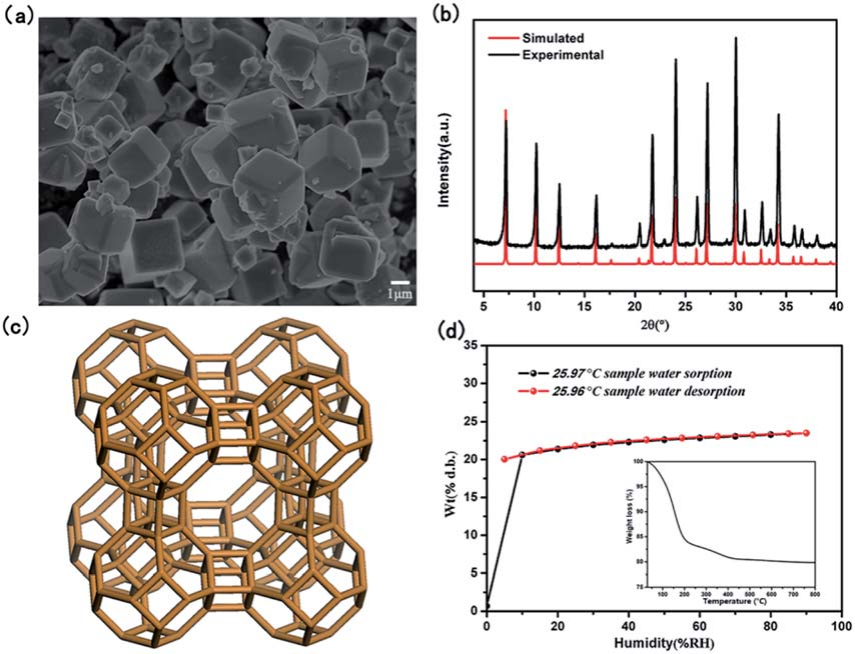
(a) SEM image of NaA zeolite; (b) PXRD patterns of synthesized and simulated NaA zeolite; (c) the topology structure of LTA; (d) water vapor sorption and desorption curves and the inset: TG analysis of as-synthesized NaA zeolite.

As shown in SEM image of Fig. 1a, the crystals of as-synthesized NaA zeolite had regular cubic morphology with the particle sizes about 1–2 μm. Its phase purity was confirmed by PXRD analysis (Fig. 1b). And the numbers of ICDD PDF cards of 4A zeolite (NaA) were 39-0223. Meanwhile, from the Fig. 1c, it showed that the structure of NaA zeolite was a simple cubic arrangement of sodalite cages connected by double four-membered rings (D4Rs) to form the larger α cages and a three-dimensional (3D) micropore structure. Thus, NaA zeolite had 8-ring channels along the directions of [100], [010] and [001], and the diameter of channel was about 4.1 Å × 4.1 Å.^27^ ICP analysis indicated that the molar ratios of Si/Al and Na/Al in the synthesized NaA zeolite were about 1. It indicated that the framework of NaA zeolite was composed by the alternation connection of AlO_4_ and SiO_4_ tetrahedra, and the Na^+^ ions should be located in the cages of NaA zeolite to balance the negative charge of framework. The TG study revealed the water content of NaA zeolite was about 21.66 wt%, which could be found from the structure between room temperature ∼ 500 °C (Fig. 1d inset). The water adsorption isotherm analysis indicated that as-synthesized NaA zeolite had a good reversible adsorption and desorption capacity for water, and the water content was about 23.49 wt% (Fig. 1d). All of these suggested that the as-synthesized NaA zeolite had an excellent water uptake capacity, and the water played a major role in water medium proton conduction to achieve efficient proton conduction property.

### Characterization of ion-exchanged NaA zeolites

**Fig. 2:**
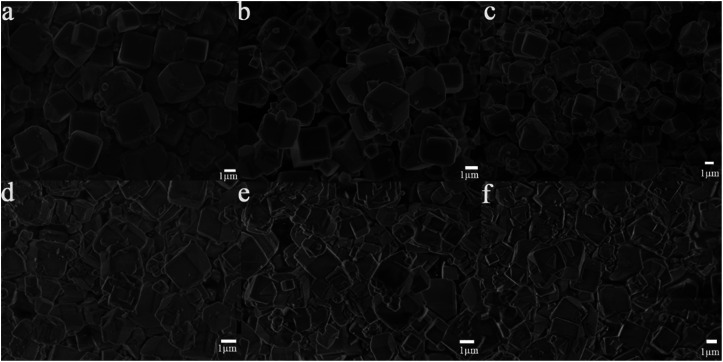
The SEM images of (a) LiA, (b) NaA, (c) KA, (d) MgA, (e) CaA and (f) SrA.

**Fig. 3:**
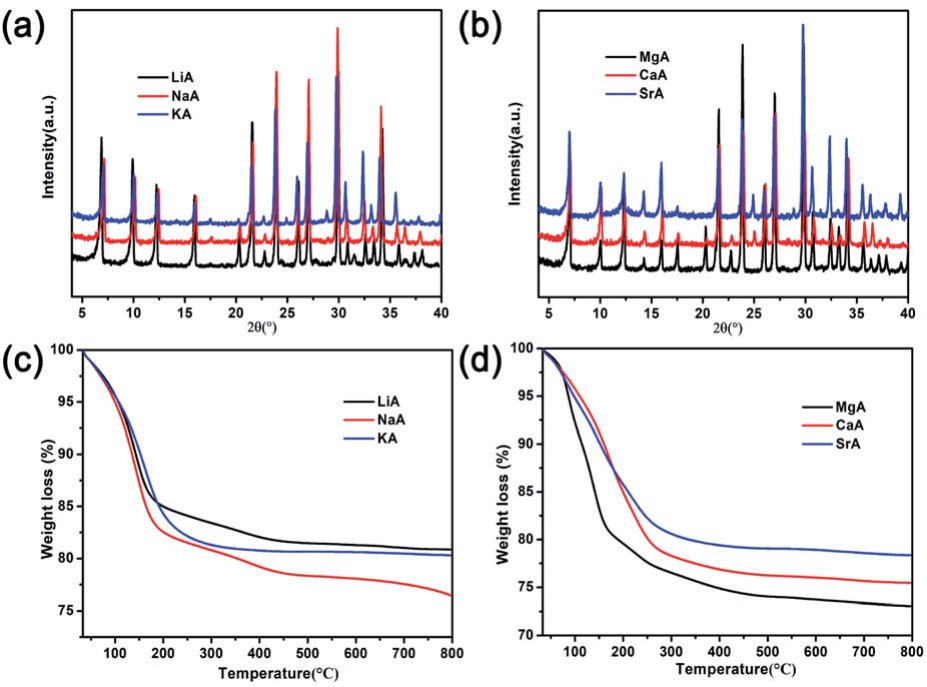
(a) and (b) PXRD patterns of different ion-exchanged of NaA zeolites; (c) and (d) TG analysis of different ion-exchanged of NaA zeolites.

In order to study the influence of guest cations in NaA zeolites on its proton conductivity, a series of other cations, such as Li^+^, K^+^, Mg^2+^, Ca^2+^, Sr^2+^ had been used to replace Na^+^ ions in NaA zeolites by ion-exchanged methods. SEM images showed that there were no obvious differences of the morphologies of guest ions substituted zeolites, and the particle sizes of them were mainly between 1 and 2 μm (Fig. 2). Most of the ion-exchanged zeolites maintained regular morphologies, but some small broken crystals could be found for MgA, CaA and SrA. Moreover, PXRD analysis indicated that the prepared LiA, KA, MgA, CaA and SrA zeolites were all pure phases (Fig. 3a and b). By calculating the relative crystallinity of as-synthesized samples based on NaA zeolite, it could be found that LiA zeolite had better crystallinity, while the relative crystallinities of other ion-exchanged zeolites had different degrees of decline. This might be because the ions of K^+^, Mg^2+^, Ca^2+^ and Sr^2+^ had bigger bulk or higher charge than Na^+^, and in ion-exchanged process, the asymmetric substitution of ions easily caused framework defects or crystallinity decline. It was noted that although LiA, KA, MgA, CaA and SrA zeolites were synthesized under the same ion-exchanged condition, their ion-exchange capacities were quite different. The molar ratio of Na/Al of the ion-exchange zeolites based on ICP analysis was shown in Fig. S1,† which indicated that LiA, KA and MgA zeolites had about 50% ion-exchange degree, lower than those of CaA and SrA zeolites (more than 80%). This meant that the high valence ions, such as Ca^2+^ and Sr^2+^, had strong ion-exchange ability, giving the lower Na/Al ratio. TG analysis in Fig. 3c and d showed the substitution of different guest ions in NaA zeolite could exhibit different water-uptake capacity. Therefore, the existence of guest ions could affect the affinity of zeolites and water molecules, which might further affect the proton conduction property of these zeolites.

### Proton conduction property of NaA zeolite

**Fig. 4:**
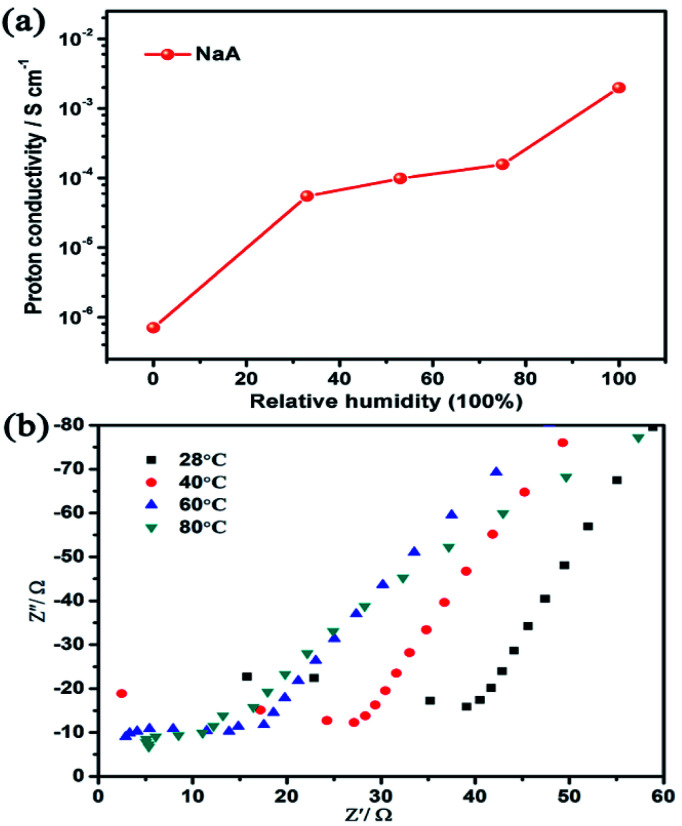
(a) The proton conductivity of NaA zeolite at different humidity and room temperature; (b) A.C impedance plots of NaA zeolite at different temperature under 100% RH.

For most materials, the realization of low temperature proton conduction generally depends on the water medium. Hence, the influence of different humidity conditions on the proton conductivity of as-synthesized NaA zeolite had been investigated. As was shown in Fig. 4a, when the sample was dried and no humidity was given, the proton conductivity of NaA was 7.04 × 10^−7^ S cm^−1^. When the relative humidity increased from 33% RH to 100% RH, the proton conductivity of NaA increased obviously, from 5.49 × 10^−5^ S cm^−1^ (33% RH) to 1.98 × 10^−3^ S cm^−1^ (100% RH) at room temperature (Fig. S2†). It suggested that high humidity environment was favourable for improving the proton conductivity of NaA zeolite. Furthermore, the influence of temperature-dependent proton conduction property was also explored from room temperature to 80 °C (Fig. 4b). The study showed the proton conductivity of as-synthesized NaA was 1.98 × 10^−3^ S cm^−1^ at room temperature. With the increasing of temperature, the proton conductivity of NaA was evidently increasing from 2.32 × 10^−3^ S cm^−1^ (28 °C) to 9.12 × 10^−3^ S cm^−1^ (80 °C) under the condition of 100% RH.

Apparently, the proton conductivity of NaA zeolite had a high response to humidity and temperature. Particularly, the proton conductivity of NaA had a marked increase of four orders of magnitude when enhanced the humidity to 100% RH. This suggested that the existence of guest water molecules in NaA zeolite could serve as a medium for proton conduction.

Under high humidity, rich water content might be suitable to form hydrogen bonding networks that could enhance the proton conduction performance. By revealing the proton conduction mechanism of NaA zeolite, *E*_a_ was given to be 0.24 eV according to the Arrhenius formula calculating, and it belonged to Grotthuss mechanism (Fig. S3†). Noting that the as-synthesized NaA zeolite had higher proton conductivity than the reported LTA zeolite film (1.1 × 10^−4^ S cm^−1^),^48^ as well as other reported zeolites, such as FAU (∼10^−4^ S cm^−1^),^35^ MCM-41 (2.9 × 10^−5^ S cm^−1^),^36^*etc.* In addition, NaA zeolite exhibited good proton conductivity compared with some reported MOFs, given in Table S1.†

### Proton conduction property of ion-exchanged and commercial LTA zeolites

**Fig. 5:**
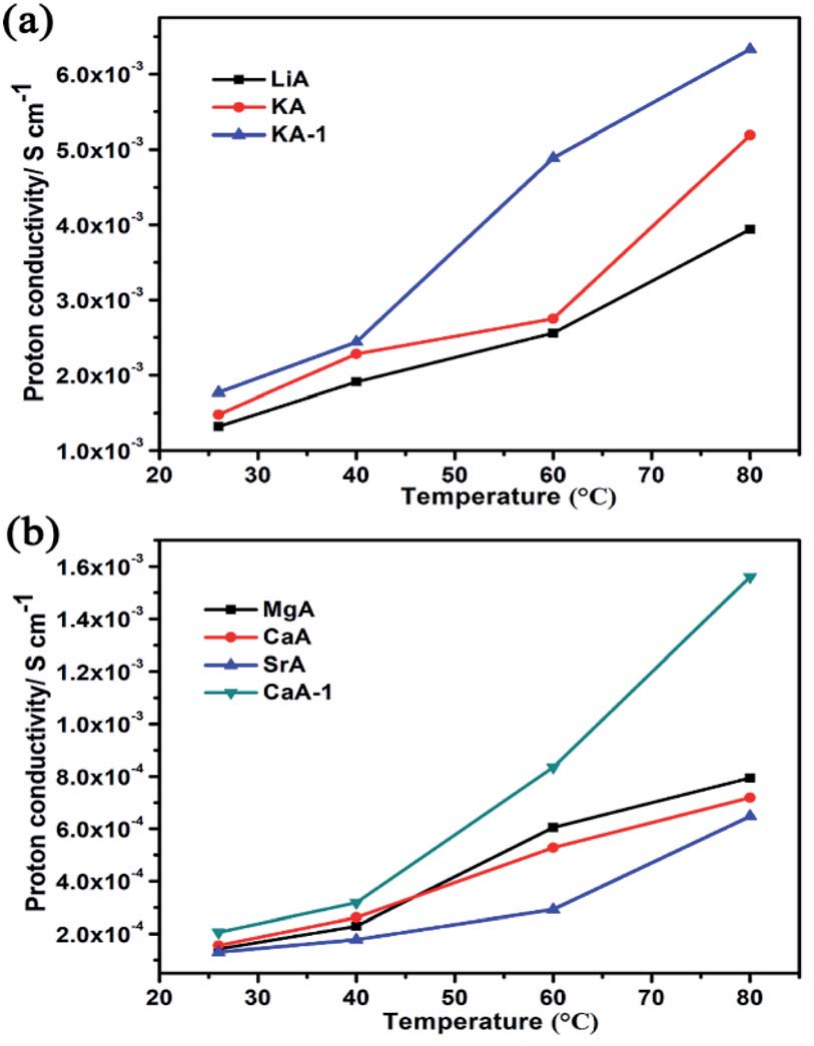
(a) The proton conductivities of LiA, KA and KA-1 at different temperature; (b) the proton conductivities of MgA, CaA, SrA and CaA-1 at different temperature.

Firstly, the influence of different ion-exchange degree of guest cations in NaA zeolite on the proton conduction had been studied. For this, KA-1 and CaA-1 zeolites with lower ion-exchange degree had been synthesized. The ratio of Na/Al for KA and KA-1 are 0.47 and 0.68, respectively, and for CaA and CaA-1 are 0.16 and 0.51, respectively, based on ICP analysis. Compared with KA and KA-1, there was no big difference in proton conductivity under low temperature (100% RH), but when the temperature increased to 80 °C, KA-1 showed higher proton conductivity (6.33 × 10^−3^ S cm^−1^) than that of KA (5.43 × 10^−3^ S cm^−1^) (Fig. 5a). The comparison of proton conductivity of CaA-1 and CaA also showed the similar regulation, the highest proton conductivity of CaA-1 could achieve 1.56 × 10^−3^ S cm^−1^ (80 °C, 100% RH), that was more significantly enhanced than CaA (7.56 × 10^−4^ S cm^−1^) (Fig. 5b). These results suggested the higher Na^+^ contents in zeolites facilitated to achieve better proton conductivity, particularly for divalent ions-containing zeolites.

Secondly, the proton conduction of different ion-exchanged NaA zeolites had been studied. As shown in Fig. 5 and S4,† with the temperature increasing, their proton conductivities increased obviously. At room temperature under 100% RH, their proton conductivities were LiA of 1.32 × 10^−3^ S cm^−1^, KA of 1.48 × 10^−3^ S cm^−1^, MgA of 1.46 × 10^−4^ S cm^−1^, CaA of 1.56 × 10^−4^ S cm^−1^ and SrA of 1.29 × 10^−4^ S cm^−1^. At high temperature of 80 °C and 100% RH, their proton conductivities had the similar regulation, the proton conductivities of LiA (3.94 × 10^−3^ S cm^−1^) and KA (5.43 × 10^−3^ S cm^−1^) that were much higher than those of MgA (8.25 × 10^−4^ S cm^−1^), CaA (7.56 × 10^−4^ S cm^−1^) and SrA (6.75 × 10^−4^ S cm^−1^). By calculating the activation energy of the as-synthesized NaA and ion-exchanged zeolites, all of them belonged to Grotthuss mechanism (Fig. S5†).

**Fig. 6:**
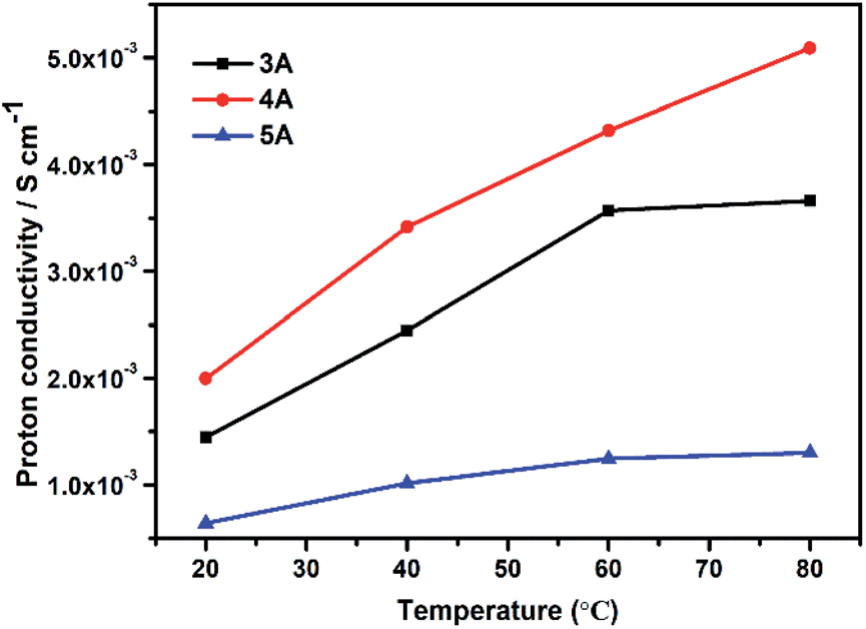
The proton conductivities of commercial 3A, 4A and 5A zeolites at different temperature.

Moreover, we also studied the proton conduction property of three commercial zeolites including 3A (KA), 4A (NaA) and 5A (CaA) for comparisons. All of their phase purity and TGs were given in Fig. S6.† The elemental analysis indicated that the Na/Al molar ratios of commercial zeolites were 0.49 in 3A, 1 in 4A and 0.20 in 5A zeolite, which were similar with those as-synthesized KA, NaA and CaA zeolites. As shown in Fig. 6, 4A zeolite possessed the best proton conductivity, the order of their proton conductivities was 4A (5.09 × 10^−3^ S cm^−1^) > 3A (3.66 × 10^−3^ S cm^−1^) > 5A (1.30 × 10^−3^ S cm^−1^) at room temperature and 80 °C (100% RH). This was consistent with the above results obtained by experimental ion-exchanged NaA zeolites.

### Effect of guest ions on proton conduction

Comparing the proton conducting performances of these ion-exchanged and commercial LTA zeolites with NaA zeolite, it can be found that various guest ions have significant effect on proton conduction property of zeolites. Among these LTA zeolites with different guest ions, NaA zeolite has the best proton conduction property. After the ion exchanging, the co-existence of Na^+^ ions and other guest ions in zeolites will lead to the different degrees of decline of proton conductivity. In addition, LTA zeolites containing monovalent guest cations (*e.g.* Na^+^, Li^+^ and K^+^) generally present better proton conduction properties than those with the divalent guest cations (*e.g.* Mg^2+^, Ca^2+^ and Sr^2+^). This means that the presence of Na^+^ ions in zeolites is most beneficial for achieving excellent proton conductivity. The reason may be that Na^+^ ions are more suitable for the pore size and pore polarity of LTA zeolite since only Na^+^ ions while not other guest ions can be used as templates to synthesize LTA zeolite, and Na^+^ ions cannot be completely exchanged by other guest ions.^27^ Meanwhile, Na^+^ ions and pore features of LTA zeolite endow NaA zeolite with good capacity of water uptake and excellent proton conductivity. When some Na^+^ ions are ion-exchanged by other guest ions, there may be interference between the coexisted two kinds of ions, resulting the decrease of proton conductivity. Such interference is more obvious when divalent cations existed in zeolites than that of monovalent cations, and the coexistence of multiple ions will increase the interference between ions that is harmful for proton conduction. Furthermore, compared with the monovalent cations, the cationic concentrations of divalent cations in zeolites will decrease and the cationic interference will increase due to their higher charges and bigger radius, resulting the lower proton conductivity.

## Conclusions

A series of LTA zeolites including NaA zeolite and ion-exchanged LiA, KA, MgA, CaA and SrA zeolites have been hydrothermally synthesized to study the effect of various guest ions on the proton conduction. The systematic investigation of the proton conduction behaviors of these zeolites demonstrates that the guest ions can influence the proton conduction property through host-guest ions interaction, cationic interference and cationic concentrations. Among various guest ions, the sodium ions are most beneficial to promote the proton conductivity of LTA zeolite. The presence of two guest ions would interfere each other to further affect the interaction between water molecules, and obstruct the proton transport. Particularly, the divalent ions (*e.g.* Mg^2+^, Ca^2+^ and Sr^2+^) are unfavourable for the proton conduction because of the low cationic concentrations and increased cationic interference. The understanding of the role of guest ions in the proton conduction will be helpful to adjust the proton conduction property and promote the applications of nanoporous materials as solid electrolytes. Of course, the influencing factors of proton conduction in zeolites may be complicated, and further research will be continued in our future work.

## Conflicts of interest

There are no conflicts to declare.

## Supplementary Material

RA-011-D0RA09917A-s001

## References

[cit1] Duan C., Hook D., Chen Y., Tong J., O'Hayre R. (2017). Energy Environ. Sci..

[cit2] Bi L., Shafi S. P., Da'as E. H., Traversa E. (2018). Small.

[cit3] Duan C., Tong J., Shang M., Nikodemski S., Sanders M., Ricote S., Almansoori A., O'Hayre R. (2015). Science.

[cit4] Mitchell-Williams T. B., Tomov R. I., Saadabadi S. A., Krauz M., Aravind P. V., Glowacki B. A., Kumar R. V. (2017). Materials for Renewable and Sustainable Energy.

[cit5] Fu X. Z., Luo J. L., Sanger A. R., Luo N., Chuang K. T. (2010). J. Power Sources.

[cit6] Tran P. D., Morozan A., Archambault S., Heidkamp J., Chenevier P., Dau H., Fontecave M., Martinent A., Jousselme B., Artero V. (2015). Chem. Sci..

[cit7] LI J., Qiao J., Lian K. (2020). Energy Storage Mater..

[cit8] Shin D. W., Guiver M. D., Lee Y. M. (2017). Chem. Rev..

[cit9] Yao Y. F., Lin Z., Li Y., Alcoutlabi M., Hamouda H., Zhang X. W., Zhang W. (2011). ACS Appl. Mater. Interfaces.

[cit10] He G. W., Li Z., Zhao J., Wang S. F., Wu H., Guiver M. D., Jiang Z. Y. (2015). Adv. Mater..

[cit11] Lu J., Lu S., Jiang S. P. (2011). Chem. Commun..

[cit12] Liu L. Y., Chen W. D., Li Y. Q. (2016). J. Membr. Sci..

[cit13] Karim M. R., Hatakeyama K., Koinumaa M., Hayam S. (2017). J. Mater. Chem. A.

[cit14] Liu L., Yao Z., Ye Y., Liu C., Lin Q., Chen S., Xiang S., Zhang Z. (2019). ACS Appl. Mater. Interfaces.

[cit15] Lim D.-W., Sadakiyob M., Kitagawa H. (2019). Chem. Sci..

[cit16] Li X.-M., Liu J., Zhao C., Zhou J.-L., Zhao L., Lia S.-L., Lan Y.-Q. (2019). J. Mater. Chem. A.

[cit17] Lai X., Liu Y., Yang G., Liu S., Shi Z., Lu Y., Luo F., Liu S. (2017). J. Mater. Chem. A.

[cit18] Leus K., Bogaerts T., De D. J., Depauw H., Hendrickx K., Vrielinck H., Van S. V., Van Der V. P. (2016). Microporous Mesoporous Mater..

[cit19] Yang F., Huang H., Wang X., Li F., Gong Y., Zhong C., Li J.-R. (2015). Cryst. Growth Des..

[cit20] Phang W. J., Jo H., Lee W. R., Song J. H., Yoo K., Kim B., Hong C. S. (2015). Angew. Chem., Int. Ed..

[cit21] Yoon M., Suh K., Natarajan S., Kim K. (2013). Angew. Chem., Int. Ed..

[cit22] Hwang S., Lee E. J., Song D., Jeong N. C. (2018). ACS Appl. Mater. Interfaces.

[cit23] Sun H., Tang B., Wu P. (2017). ACS Appl. Mater. Interfaces.

[cit24] Yang F., Xu G., Dou Y., Wang B., Zhang H., Wu H., Zhou W., Li J.-R., Chen B. (2017). Nat. Energy.

[cit25] Guo Y., Jiang Z., Ying W., Chen L., Liu Y., Wang X., Jiang Z. J., Chen B., Peng X. (2018). Adv. Mater..

[cit26] Li X.-M., Dong L.-Z., Li S.-L., Xu G., Liu J., Zhang F.-M., Lu L.-S., Lan Y.-Q. (2017). ACS Energy Lett..

[cit27] Xu R. , Pang W., Yu J., Huo Q. and Chen J.. Chemistry of zeolites and related porous materials: synthesis and structure, Wiley & Sons (Asia) Pte Ltd, Singapore, 2007

[cit28] Čejka J. , Corma A. and Zones S., Zeolites and catalysis: Synthesis Reactions and Applications, Wiley-VCH Velag GmbH & Co., KGaA, Weinheim, 2010

[cit29] Wang C., Liu X., Demir N. K., Chen J. P., Li K. (2016). Chem. Soc. Rev..

[cit30] Weckhuysen B. M., Yu J. (2015). Chem. Soc. Rev..

[cit31] Li J., Corma A., Yu J. (2015). Chem. Soc. Rev..

[cit32] Holm M. S., Taarning E., Egeblad K., Christensen C. H. (2011). Catal. Today.

[cit33] Mikhailenko S. D., Kaliaguine S., Ghali E. (1997). Microporous Mater..

[cit34] Felice V., Ntais S., Tavares A. C. (2013). Microporous Mesoporous Mater..

[cit35] Felice V., Tavares A. C. (2011). Solid State Ionics.

[cit36] McKeen J. C., Yan Y. S., Davis M. E. (2008). Chem. Mater..

[cit37] Franke M. E., Simon U. (2004). ChemPhysChem.

[cit38] Han W., Kwan S. M., Yeung K. L. (2012). Chem. Eng. J..

[cit39] Munavalli B. B., Kariduraganavar M. Y. (2019). Electrochim. Acta.

[cit40] Krathumkhet N., Vongjitpimol K., Chuesutham T., Changkhamchom S., Phasuksom K., Sirivat A., Wattanakul K. (2018). Solid State Ionics.

[cit41] Zhang Q., Chen H., Wu T., Jin T., Pan Z., Zheng J., Gao Y., Zhuang W. (2017). Chem. Sci..

[cit42] Stirnemann G., Wernersson E., Jungwirth P., Laage D. (2013). J. Am. Chem. Soc..

[cit43] Tielrooij K. J., Garcia-Araez N., Bonn M., Bakke H. J. (2010). Science.

[cit44] Smith J. D., Saykally R. J., Geissler P. L. (2007). J. Am. Chem. Soc..

[cit45] Miner E. M., Park S. S., Dincă M. (2019). J. Am. Chem. Soc..

[cit46] Sun Y., Yan Y., Wang Y., Li Y., Li J., Yu J. (2015). Chem. Commun..

[cit47] Mu Y., Wang Y., Li Y., Li J., Yu J. (2015). Chem. Commun..

[cit48] Wu G., Zhang H., Zhou J., Huang A., Wan Q. (2013). J. Mater. Chem. C.

