# Supplementary material for: The effect of guest cations on proton conduction of LTA zeolite
Source: RSC Adv. 2021 Jan 29;11(10):5393–8. doi: 10.1039/d0ra09917a (PMC8694635; doi:10.1039/d0ra09917a)
Supplement: RA-011-D0RA09917A-s001 [file RA-011-D0RA09917A-s001.pdf]

Electronic Supplementary Material (ESI) for RSC Advances.
This journal is © The Royal Society of Chemistry 2021

Supporting Information

The effect of guest cations on proton conduction of LTA zeolite

Huaizhong Shi, Jiani Zhang and Jiyang Li*

State Key Laboratory of Inorganic Synthesis & Preparative Chemistry, College of Chemistry, Jilin University, Changchun 130012, P. R. China.

Email: lijiyang@jlu.edu.cn

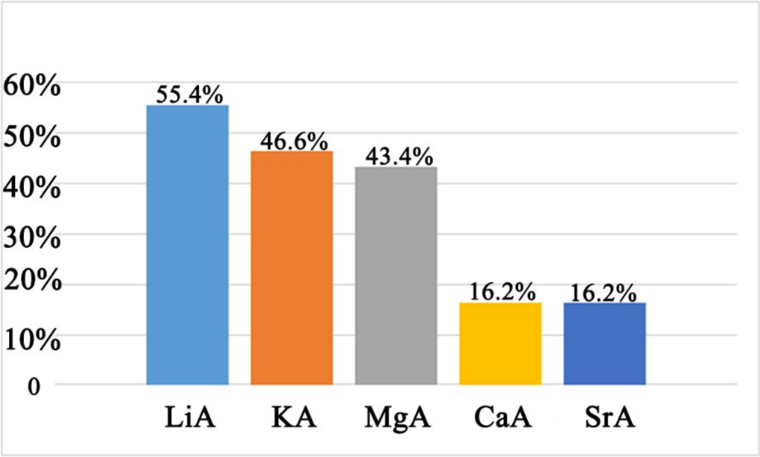

Fig. S1 The mol ratio of Na/Al in ion-exchanged zeolites.

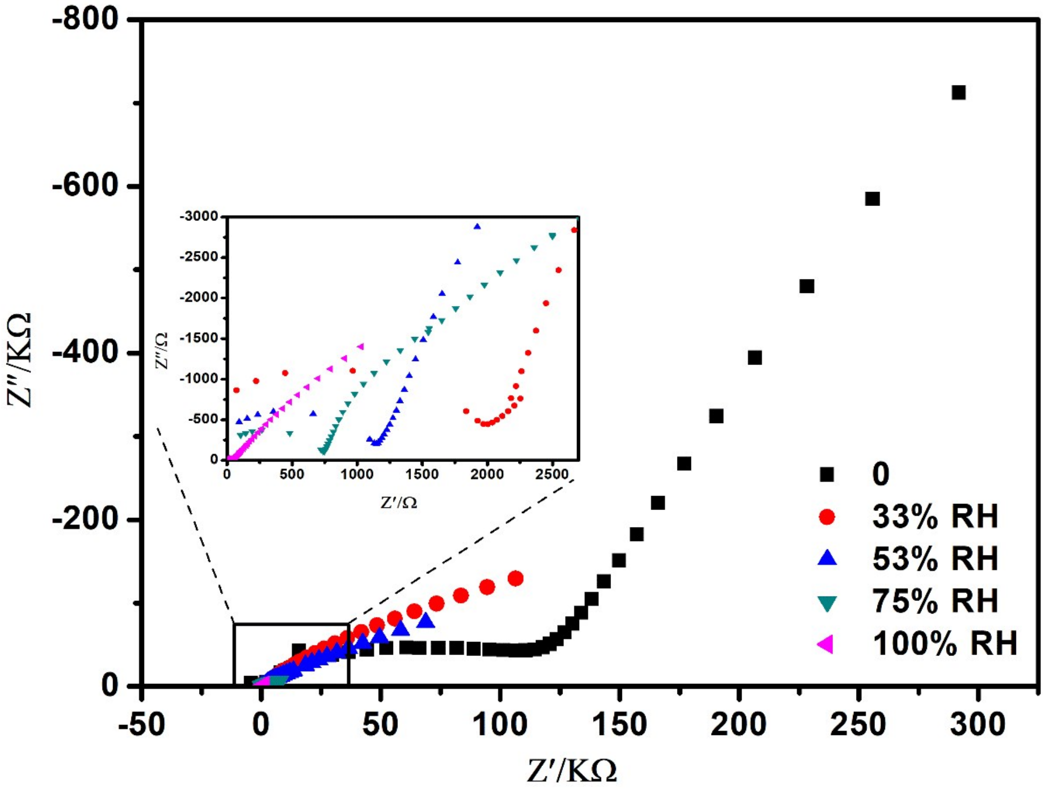

Fig. S2 A.c impedance plots of humidity-dependent of NaA zeolite at room temperature.

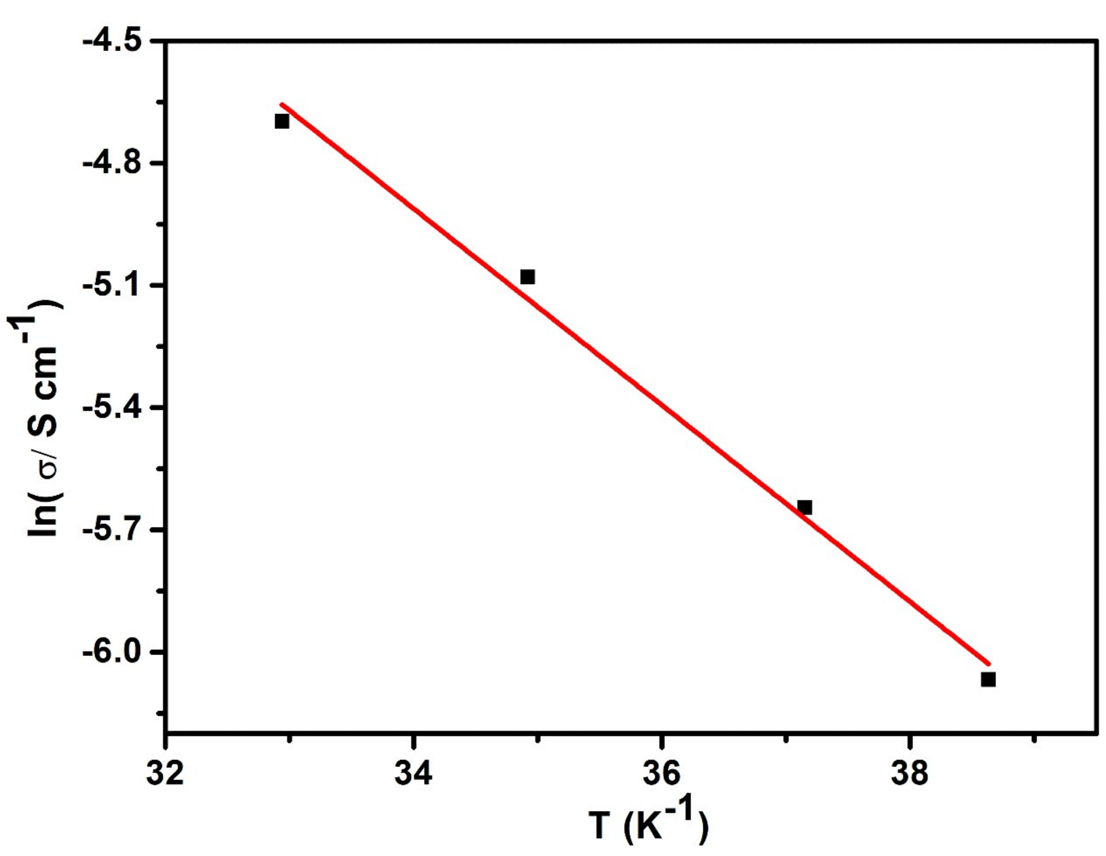

Fig. S3 The fitting curve of Ea (Ea=0.24 eV) by Arrhenius equation.

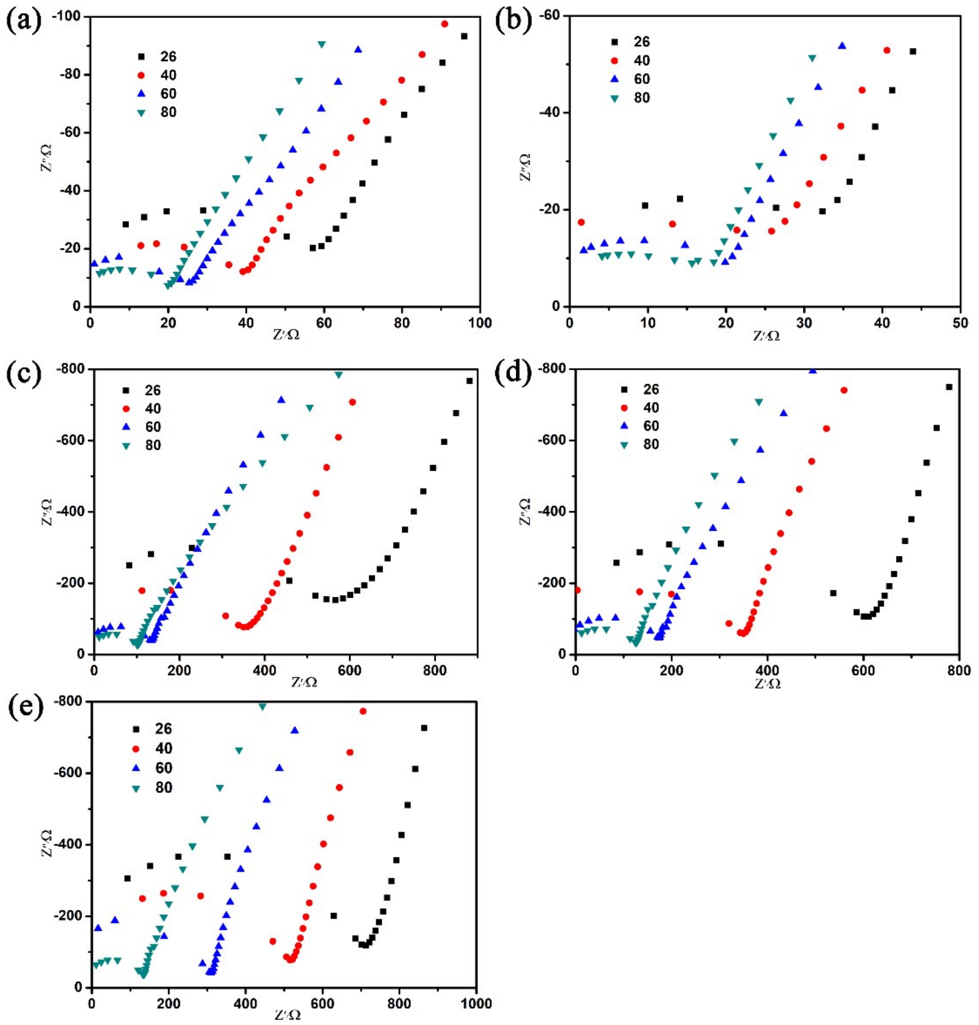

Fig. S4 A.c impedance plots of zeolites at different temperature under 100% RH (a) LiA, (b) KA, (c) MgA, (d) CaA and (e) SrA.

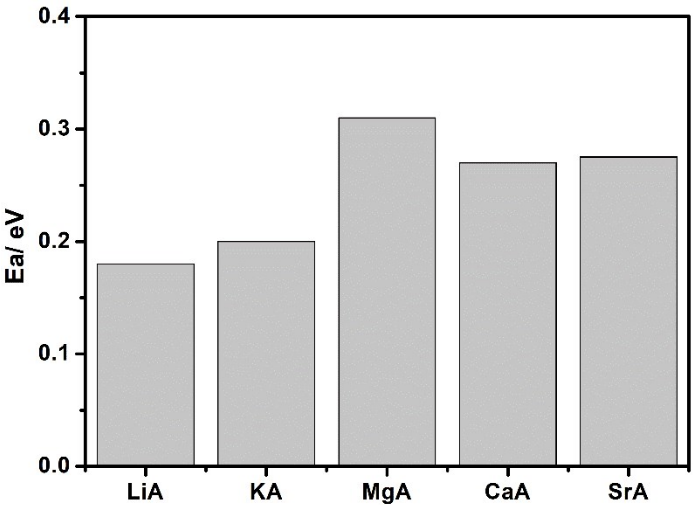

Fig. S5 The Ea of ion-exchanged zeolites (a) LiA, (b) KA, (c) MgA, (d) CaA and (e) SrA.

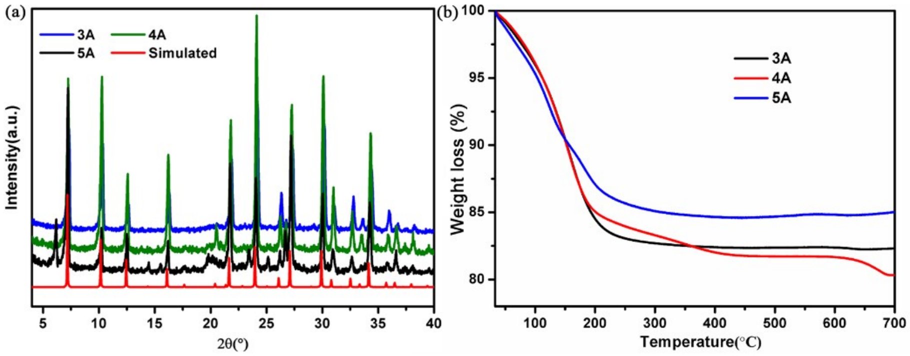

Fig. S6 (a) PXRD and (b) TG of the commercial zeolites of 3A, 4A and 5A.

Table S1. Comparison of proton conductivities of some reported MOFs.

Material	Proton conductivity (S cm^{-1})	Temperature (°C) and RH	Activation energy (eV)
$[Fe(ox)(H_2O)_2]$ [1]	1.3×10^{-3}	25 °C, 98% RH	0.37
$(NH_4)_2(H_2adp)[Zn_2(ox)_3]$ [2]	0.8×10^{-2}	25 °C, 98% RH	0.63
$(N_2H_5)[CeEu(ox)_4(N_2H_5)]\cdot 4H_2O$ [3]	3.42×10^{-3}	25 °C, 100% RH	0.10
$Zr_6O_4(OH)_6(p\text{-}BDC)_5$ (UiO-66-6) [4]	6.93×10^{-3}	25 °C, 100% RH	0.22
Im@MOF-808 [5]	3.45×10^{-2}	65 °C, 99% RH	0.25
$Mg_2(H_2O)_4(H_2DBDP)\cdot H_2O$ (PCMOF10) [6]	3.55×10^{-2}	70 °C, 98% RH	0.40
This work	9.12×10^{-3}	80 °C, 100% RH	0.24

Reference

[1] T. Yamada, M. Sadakiyo and H. Kitagawa, *J. Am. Chem. Soc.*, 2009, **131**, 3144–3145.

[2] M. Sadakiyo, T. Yamada and H. Kitagawa, *J. Am. Chem. Soc.,* 2009, **131**, 9906–9907.

[3] K. Zhang, X. Xie, H. Li, J. Gao, L. Nie, Y. Pan, J. Xie, D. Tian, W. Liu, Q. Fan, H. Su, L. Huang and W. Huang, *Adv. Mater.,* 2017, 29, 1701804.

[4] J. M. Taylor, S. Dekura, R. Ikeda and H. Kitagawa, *Chem. Mater.,* 2015, **27**, 2286–2289.

[5] H. B. Luo, Q. Ren, P. Wang, J. Zhang, L. F. Wang and X. M. Ren, *ACS Appl. Mater. Interfaces,* 2019, **11**, 9164–9171.

[6] P. Ramaswamy, N. E. Wong, B.S. Gelfand and G. K. Shimizu, *J. Am. Chem. Soc.,* 2015, **137**, 7640–7643.
